# Direct Conversion of Human Urine Cells to Neurons by Small Molecules

**DOI:** 10.1038/s41598-019-53007-6

**Published:** 2019-11-13

**Authors:** Guosheng Xu, Feima Wu, Xiaotong Gu, Jiaye Zhang, Kai You, Yan Chen, Anteneh Getachew, Yuanqi Zhuang, Xiaofen Zhong, Zuoxian Lin, Dongsheng Guo, Fan Yang, Tingcai Pan, Hongcheng Wei, Yin-xiong Li

**Affiliations:** 1 Institute of Public Health, Guangzhou Institutes of Biomedicine and Health, Chinese Academy of Sciences, Guangzhou, China; 2 Guangdong Provincial Key Laboratory of Biocomputing, Guangzhou Institutes of Biomedicine and Health, Chinese Academy of Sciences, Guangzhou, China; 3 Key Laboratory of Regenerative Biology, South China Institute for Stem Cell Biology and Regenerative Medicine, Guangzhou Institutes of Biomedicine and Health, Chinese Academy of Sciences, Guangzhou, China; 4 University of Chinese Academy of Sciences, Beijing, China; 5 Guangdong Provincial Key Laboratory of Stem Cell and Regenerative Medicine, Guangzhou, China; 6 Guangzhou Blood Center, Guangzhou, China; 7 Department of Gastroenterology, The First Affiliated Hospital of Jinan University, Guangzhou, China; 8 Guangzhou Regenerative Medicine and Health Guangdong Laboratory, Guangzhou, China

**Keywords:** Cellular neuroscience, Cellular neuroscience, Transdifferentiation, Transdifferentiation, Transdifferentiation

## Abstract

Transdifferentiation of other cell type into human neuronal cells (hNCs) provides a platform for neural disease modeling, drug screening and potential cell-based therapies. Among all of the cell donor sources, human urine cells (hUCs) are convenient to obtain without invasive harvest procedure. Here, we report a novel approach for the transdifferentiation of hUCs into hNCs. Our study demonstrated that a combination of seven small molecules (CAYTFVB) cocktail induced transdifferentiation of hUCs into hNCs. These chemical-induced neuronal cells (CiNCs) exhibited typical neuron-like morphology and expressed mature neuronal markers. The neuronal-like morphology revealed in day 1, and the Tuj1-positive CiNCs reached to about 58% in day 5 and 38.36% Tuj1+/MAP2+ double positive cells in day 12. Partial electrophysiological properties of CiNCs was obtained using patch clamp. Most of the CiNCs generated using our protocol were glutamatergic neuron populations, whereas motor neurons, GABAergic or dopaminergic neurons were merely detected. hUCs derived from different donors were converted into CiNCs in this work. This method may provide a feasible and noninvasive approach for reprogramming hNCs from hUCs for disease models and drug screening.

## Introduction

Despite the huge demand for human neuronal cells (hNCs) in neural disease modeling, drug screening, and cell-based therapies, the invasive collection of primary neuronal cells limits the supply of hNCs^1^. To expand the sources of hNCs, embryonic stem cells (ESCs) and induced pluripotent stem cells (iPSCs) have been differentiated into neurons *in vitro*^2–7^. However, current methods for generating neurons by differentiation of ESCs or iPSCs are time-consuming and inefficient. Moreover, teratomas introduces potential risk for clinical applications.

Transdifferentiation is a process that one type of differentiated somatic cells directly switches into another cell type without passing through an intermediate pluripotent stage. This provides an alternative approach for generating desired cell types. Moreover, lineage reprogramming can quickly manipulate cell fate and obtain target cell types, thereby avoiding the risk of teratomas. Enforced expression of transcription factors, which determine cell fate, could induce lineage conversion between different cell types. Combinatorial expression of neuronal-lineage-specific transcription factors could directly convert fibroblasts or other somatic cells into neurons^8–10^. However, using ectopic transgenes to induce lineage conversion still faces safety concerns.

As an alternative, small molecules approach shows advantages in transdifferentiation. Small molecules approach has been widely used in cell differentiation, cell reprogramming, and cell-cell transdifferentiation. These molecules can facilitate exogenous gene-driven transdifferentiation^11–14^. Moreover, previous studies shown that a complete chemical induction medium is sufficient to reprogram a cell lineage, without integrating exogenous genes^15–18^. This shown that small molecules method is non-immunogenic and cost-effective.

Moreover, the small molecules are easy to synthesize, manipulate, and standardize. Hence, small molecules inducing cell-lineage reprogramming might be a better method in producing neuronal cells.

A key issue in direct neuronal reprogramming is the selection of the starting cell type. The ideal cell source should be easy to access. Previous studies shown that astrocytes^19,20^, glial cells^21^, and fibroblasts^16,19,22,23^ could be directly converted into neurons through small molecules induction without integrating exogenous genes. However, invasive surgical procedures are necessary to harvest these cells.

In contrast, human urine cells (hUCs) isolated from voided urine avoids invasive surgical biopsy procedures^24–27^. Thus, hUCs is a convenient material for lineage reprogramminge. In this study, we used hUCs for neuronal transdifferentiation. We tested a variety of small molecules targeting signaling pathways important for neurogenesis and neuronal differentiation and then converted hUCs into neurons by small molecules inducing cell-lineage reprogramming, without integrating exogenous genes. The results shown that applying a combination of seven small molecules to human urine cells could induce the hUCs into neurons within a few days.

## Results

### CAYTFVB seven small molecules could convert human urine cells into neurons

Approximately 2000 to 7000 cells would detach along with the urinary tract daily^26^. These cells in urine are still functional, which can be collected and expanded *in vitro*. We isolated hUCs (UCX001) from the urine sample of a healthy 32-year-old male donor using the protocol reported previously^27^ (Supplementary Fig. S1A). To test whether the hUCs contained any neural stem cells (NSCs) or neuronal cells, we performed immunostaining assay with Nestin, PAX6, Tuj1, MAP2, DCX, NeuN, and GFAP antibodies. Results showed that neither NSCs nor neurons were found in the hUCs (Supplementary Fig. S1B).

Recent studies have shown that small molecules which activate Wnt signaling and block TGF-β signaling could improve the efficiency of neuronal differentiation of human ES and iPS cells^28,29^. Furthermore, activation of Wnt and blocking of TGF-β signaling was also an important mechanism for direct neuronal conversion using small molecules^16,19^. Thus, we hypothesized that combining TGF-β signaling inhibitor, Wnt signaling activator, and other small molecules could drive the conversion of hUCs into neuronal cells.

To achieve this, we treated the hUCs with CHIR99021 (an activator of Wnt signaling) and A8301(a blocker of TGF-β signaling) in a basic neuronal induction medium and some neuron-like cells were observed at day 10 (Supplementary Fig. S2A). However, the induced cells were not typical neuronal morphology and the inductive efficiency was very low. To solve this problem, we screened other small molecules that may facilitate the conversion of hUCs into neurons. A neuronal induction medium which contains a cocktail of CHIR99021 (C), A8301 (A), Y27632 (Y), TTNPB (T), and Forskolin (F) was applied to hUCs. This combination introduces a rapid morphological change of the cells. The neuron-like cells can be observed as early as 12 h, and a significant fraction of cells exhibited neuron-like morphology with small, compact cell bodies which monopolar or bipolar projection occurred after 10 to 14 days (Supplementary Fig. S2A).

Moreover, the expression of neuronal master genes *ASCL1* and *TUBB3* were up-regulated only 1 day after CAYTF treatment (Supplementary Fig. S2B). These findings suggested that the chemical cocktail CAYTF promoted the transdifferentiation of the hUCs into neuronal fate. However, these cells were still primitive neuron-like morphology and not typical mature neuronal morphology, suggesting a partial conversion with the current protocol.

**Figure 1:**
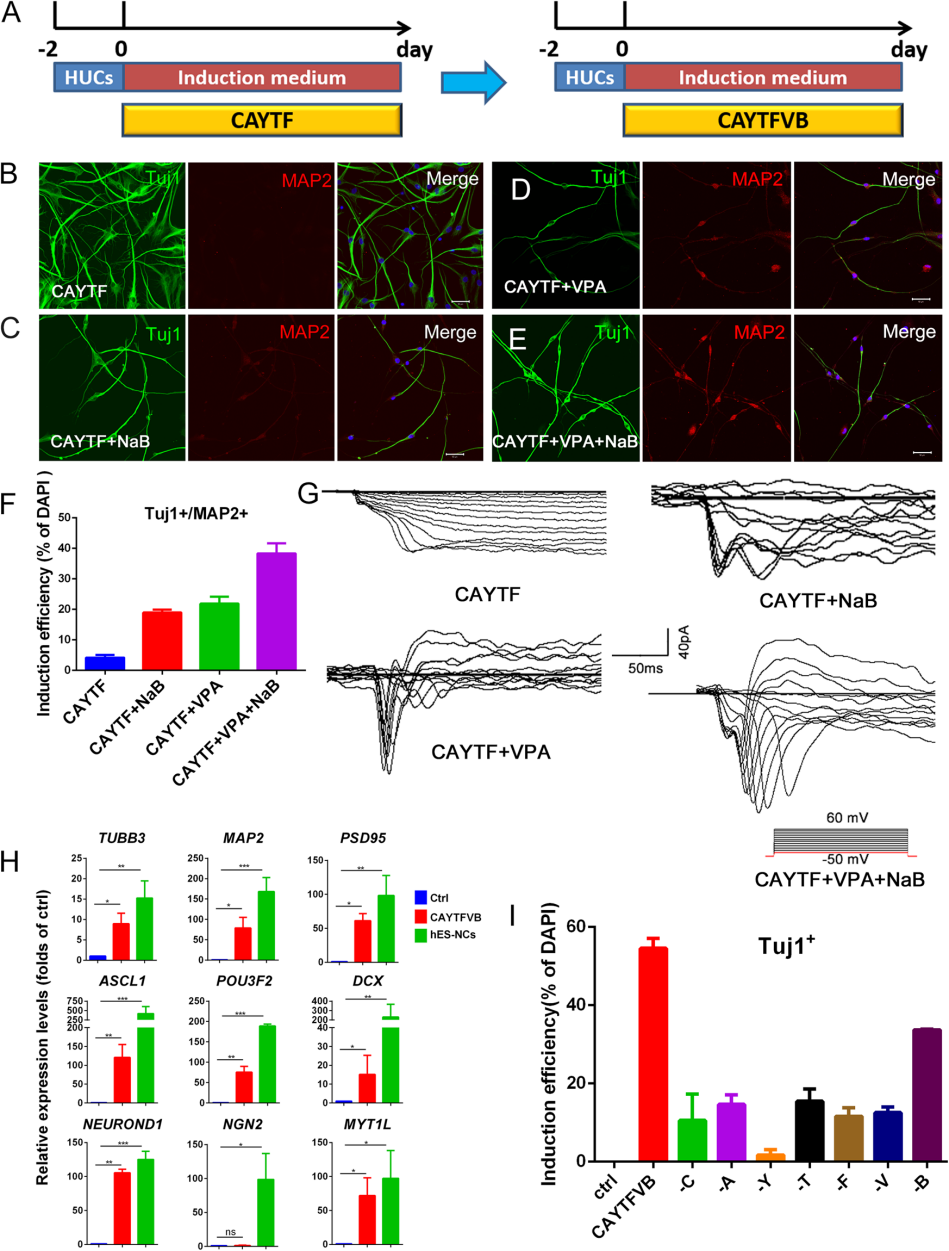
CAYTFVB seven small molecules could convert human urine cells into neurons. (**A**) Scheme of induction procedure. C, CHIR99021; A, A8301; Y, Y-27632; T, TTNPB; F, Forskolin; V, VPA; B, NaB. (**B**–**E**) Immunofluorescence staining analysis showed that VPA and NaB promote the generation of Tuj1+/MAP2+ neuronal cells. Cells were treated with CAYTF, CAYTF + NaB, CAYTF + VPA, or CAYTF + VPA + NaB respectively, immunofluorescence staining was performed at day 12. Scale bars, 50 μm. (**F**) Quantification of Tuj1+/MAP2 + cells. Cells were counted 12 days post chemical treatments. (means ± SEM, n = 20 random selected × 20 fields from triplicate samples). (**G**) Voltage-clamp recordings of cells 12 days post chemical treatments. Cells were depolarized from −50 mV to 60 mV in 10 mV increments. (**H**) Neuronal genes were upregulation at day 7 during chemical induction. hUCs were treated with CAYTFVB for 7 days. hUCs (no treatment) were used as negative control and all sample data was normalized to that of hUCs, which was considered as 1. hES derived neurons were used as positive control. Data of three independent experiment were shown as means ± SEM. Statistical assessment of the differences was performed by one-way ANOVA compared to negative control group. (* p ≤ 0.05, ** p ≤ 0.01, ***p ≤ 0.001, ns = not significant). (**I**) Withdrawal of any small molecule from CAYTFVB cocktail resulted in a reduction of the induction efficiency. hUCs were treated with indicated chemical for 5 days. The percentage of Tuj1-positive neuronal cells represent the induction efficiencies. (means ± SEM, n = 20 random selected × 20 fields from triplicate samples).

Thus, additional chemicals to promote neuronal conversion was screened. Considering that cell fate conversion was accompanied by remodeling of the epigenome, we added small molecules that modulate epigenetic enzymes into the neuronal induction medium. As a result, the additional epigenetic state-manipulating small molecules VPA (V, valproic acid) and NaB (B) in the CAYTF cocktail (Fig. 1A) improved the efficiency of generating Tuj1^+^/MAP2^+^ neuron-like cells significantly, i.e., the percentage of Tuj1^+^/MAP2^+^ cells observed by applying CAYTF, CAYTF + NaB, CAYTF + VPA, or CAYTF + VPA + NaB was 4.18%, 18.99%, 21.89%, and 38.36% at day 12, respectively (Fig. 1B–F). Furthermore, the whole-cell patch-clamp analysis was conducted to identify these cells. Fast inward sodium current and voltage-gated potassium currents were measured on the cells which been applied CAYTF + VPA + Na cocktail, while the cells with CAYTF did not possess these basic electrophysiological properties of neurons (Fig. 1G). In summary, the seven small molecules cocktail CAYTFVB provides a better result (Fig. 1A).

In the first protocol, the basic neuronal induction medium contained 8 components, including B27, ITS, EGF, Nico, FGF10, Glutamax, HGF, and N2 (Supplementary Table S1). To optimized the basic neuronal induction medium, each of these components were removed from the first neuronal induction medium used in this work (NM1). Interestingly, in the absence of B27 and Glutamax from NM1, the efficiency of Tuj1^+^ cells generation was significantly improved (Supplementary Fig. S3A, B). Moreover, the removal of all the 8 components can still generate Tuj1^+^ neuron-like cells, suggesting that small molecules CAYTFVB alone was enough to induce the conversion of hUCs into neurons (Supplementary Fig. S3A, B). Thus, we removed B27 and Glutamax from NM1 basic neuronal induction medium and formed a new basic medium NM2 (Supplementary Table S1) for the second round of the factor deduction test. In the second-round test, the efficiency of Tuj1^+^ cells generation was further improved without N2, while the absence of HGF and ITS made no change on the efficiency (Supplementary Fig. S3C). Thus, an optimized basic neuronal induction medium NM3 containing EGF, Nico, and FGF10 was produced (Supplementary Table S1).

In order to further characterize whether those CAYTFVB reprogrammed cells expressed more neuronal specific genes, RT-PCR was performed at Day 7 after CAYTFVB induction. hES derived neurons(hES-NCs) were used as the positive control, and the negative control was untreated hUCs. The expressions of most neuronal specific genes including *TUBB3, MAP2, PSD95, ASCL1, POU3F, DCX, NEUROND1 and MYT1L* were increased from 10 to 100 folds compared to the negative control, except the expression of *NGN2* (Fig. 1H). Comparing to the hES-NCs positive control, the expressions of *NEUROND1, MYT1L, PSD95* and *TUBB3* reached about two third of the positive control; followed with the *MAP2* expression level was half of the positive control, while it was one third or less for *POU3F, ASCL1* and *DCX*.

The importance of each molecule in CAYTFVB was further examined. It was observed that the absence of any of these small molecules resulted in a reduction of the induction efficiency (Fig. 1I).

In summary, the culture medium containing seven small molecules CAYTFVB in NM3 was the optimized choice for inducing neuronal conversion from hUCs. This combination was used in further experiments.

### Generation of mature neurons by using a two-stage induction protocol

Even though the hUCs can be converted to neuron efficiently with the above protocol, a significant number of induced neuronal cells began to die 12 days after applied CAYTFVB, and most induced neurons died at 16–20 days (data are not shown). Previous studies was that prolonged exposure of VPA and NaB increased cell death^20^, so the VPA and NaB was removed 7 days after applying CAYTFVB. Furthermore, recent studies have shown that a metabolic switch from higher glycolytic metabolism to more OxPhos occurs in the direct reprogramming of fibroblasts or astrocytes into neurons^30,31^, which suggests excessive oxidative stress may lead to cell death during this metabolic switch. As a counter measurement, add in antioxidant may protect the neurons from oxidative stress^32^. In this case, vitamin C (VC) shown the ability to significantly improve the survival and maturation of the induced neuronal cells.

**Figure 2:**
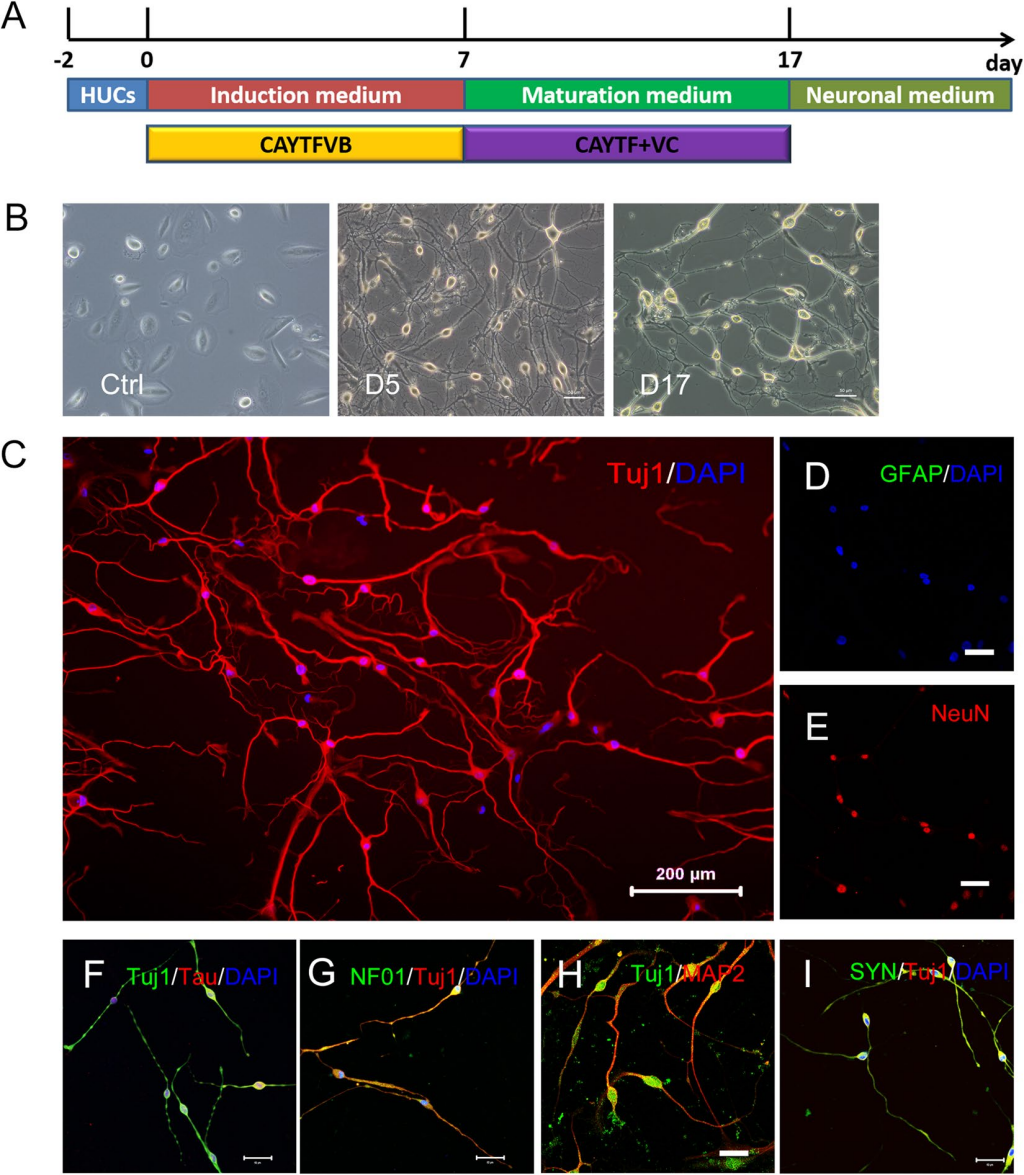
VC promotes the survival and maturation of the induced neuronal cells. (**A**) Scheme of induction procedure. Initial urine cells were seeded onto plates and cultured in urine cells medium. After 2 days, cells were transferred into neuronal induction medium with chemical cocktails CAYTFVB for 7 days, and then cells were switched to neuronal maturation medium with CAYTF + VC for another 10 days. Afterwards, cells were cultured with neuronal medium. C, CHIR99021; A, A8301; Y, Y-27632; T, TTNPB; F, Forskolin; V, VPA; B, NaB; VC, vitamin C. (**B**) Bright-field image of control hUCs (left) or CiNCs at day 5 (middle) and day 17 (right). Scale bars, 50 μm. (**C**) Induced cells display typical neuronal morphologies and express Tuj1 at day 17. Scale bars, 200 μm. (**D**–**I**) Immunofluorescence staining of CiNCs at day 17 with antibodies against the indicated markers: GFAP, astrocyte marker; NeuN, Tau, NF-01, MAP2 and SYN, mature neuronal markers. Scale bars, 50 μm.

According to the above finding, a two-stage induction protocol was established. The first stage was applying CAYTFVB to culture hUCs for 7 days. Then, change the medium to the neuron maturation medium, which contained small molecules CAYTF + VC and extra neurotropic factors (BDNF, GDNF, and NT3) for 10 days. Afterwards, culture medium changed to neuronal medium (Fig. 2A). Within 2 weeks, an increasing number of cells displayed more extensively extended neurite outgrowth consisting of multiple neurites with intricate branching and connected joint points (Fig. 2B,C). Besides, Tuj1 and mature neuronal marker MAP2, NeuN, Tau, synapsin (SYN), and NF-01 shown a positive result in immunostaining test at day 17, while it was negative for astrocytes marker GFAP (Fig. 2C–I). After observing these characteristics, these cells were believed to be CiNCs.

The morphology changes of these CiNCs which induced according to the two-stage induction protocol was recorded (Supplementary Fig. S4). The axon-like component appears within 12 hours after applying CAYTFVB, and the typical neuron morphology can be observed as early as 3 days (Supplementary Fig. S4A). when cells were cultured with neuron maturation medium more dendrite-like components occur on day 16 (Supplementary Fig. S4A-B). After removal of the small molecules, the CiNCs maintained the neural morphology and no more visual difference at day 30 (Supplementary Fig. S4A-B). The neuron marker Tuj1 was observed in the whole induction process (Supplementary Fig. S4B). Apart from Tuj1, other neuron markers including Tau, NF-01 and SYN were also presented at day 30 (Supplementary Fig. S4C). These evidences indicated the CiNCs maintained their identity after the removal of the small molecules.

Afterward, a test was conducted to discover whether the process converts hUCs to neurons directly, bypasses the neuronal progenitor stage. The expression of neuronal progenitor genes FoxG1, Nestin, Pax6, or Sox2 was absent during the CiNCs induction procedure, verified by using qRT-PCR and immunostaining analysis (Supplementary Fig. S5A–E). Thus, hUCs converted into neurons directly without the neuronal progenitor stage.

The hUCs derived from different donors was used to generate the CiNCs to verify the universal capability of this protocol. The hUCs from three different healthy donors (Supplementary Table S2) was isolated (UCX003, UCX004, and UCX007), and treated with the protocol described above. The results showed that all these three human urine cell lines could be converted into CiNCs. Neuronal markers Tuj1, MAP2, and NeuN were observed using immunofluorescent staining (Supplementary Fig. S6A–C) after 17 days of induction. These results demonstrated that the protocol developed in this study was a universal approach to convert hUCs into CiNCs.

### Induced cells are glutamatergic neurons

**Figure 3:**
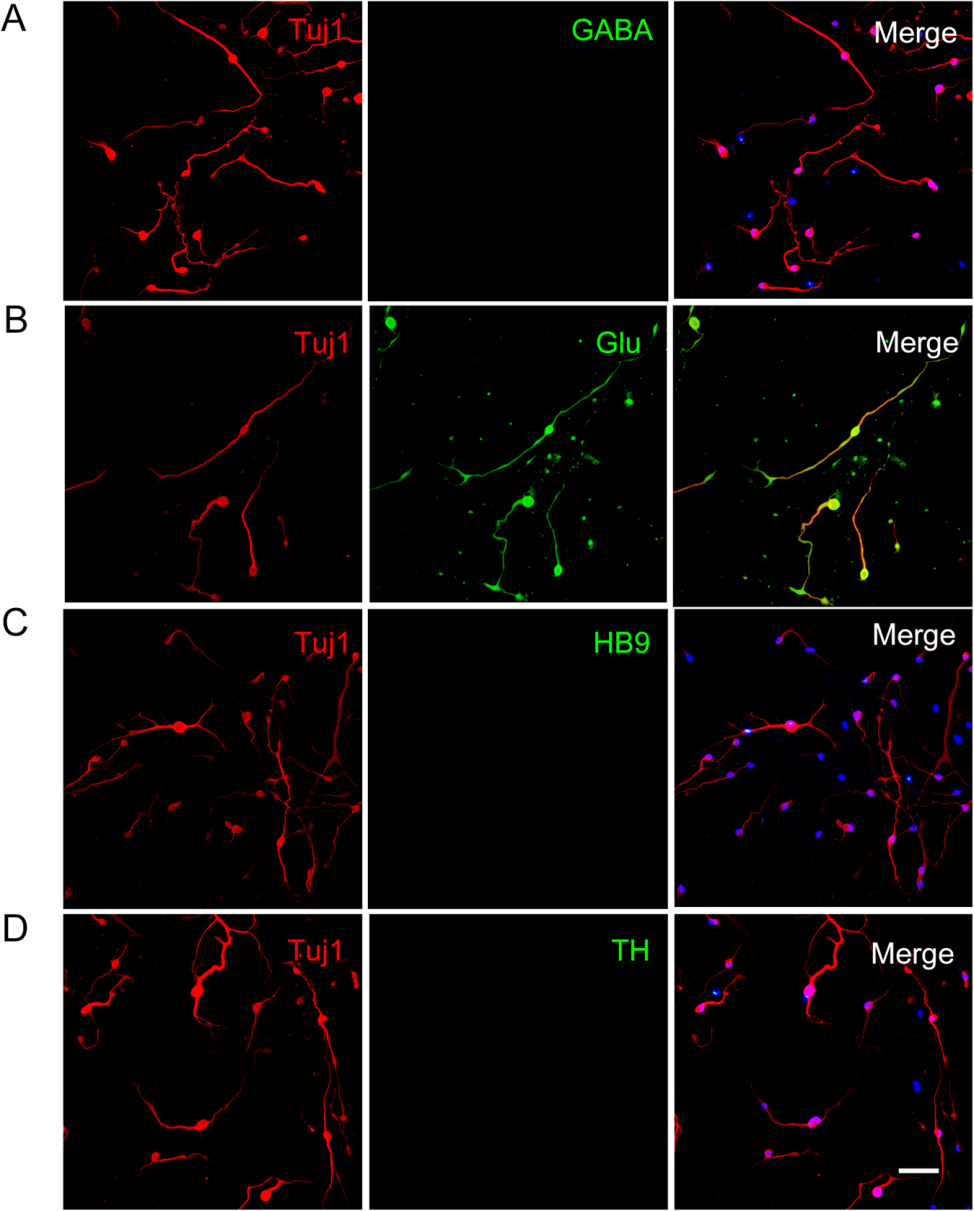
Induced cells are glutamatergic neurons. (**A**–**D**) Immunofluorescence staining of CiNCs for subtype-specific neuronal markers. The neuron type was characterized at day 17. Induced neurons express glutamatergic marker (Glu), while other neuron type markers including GABA, HB9 and TH are rarely detected. Scale bars, 100 μm.

To further understand the conversion of hUCs to neurons, a specific type of neurons obtained using our induced protocol was tested using immunostaining assay with GABA, Glu, HB9, and TH after 17 days of induction. Results showed that most of the Tuj1-positive cells were glutamatergic neurons, while motor neurons, GABAergic or dopaminergic neurons, were merely detected (Fig. 3A–D).

### Induced cells possess partial electrophysiological properties

**Figure 4:**
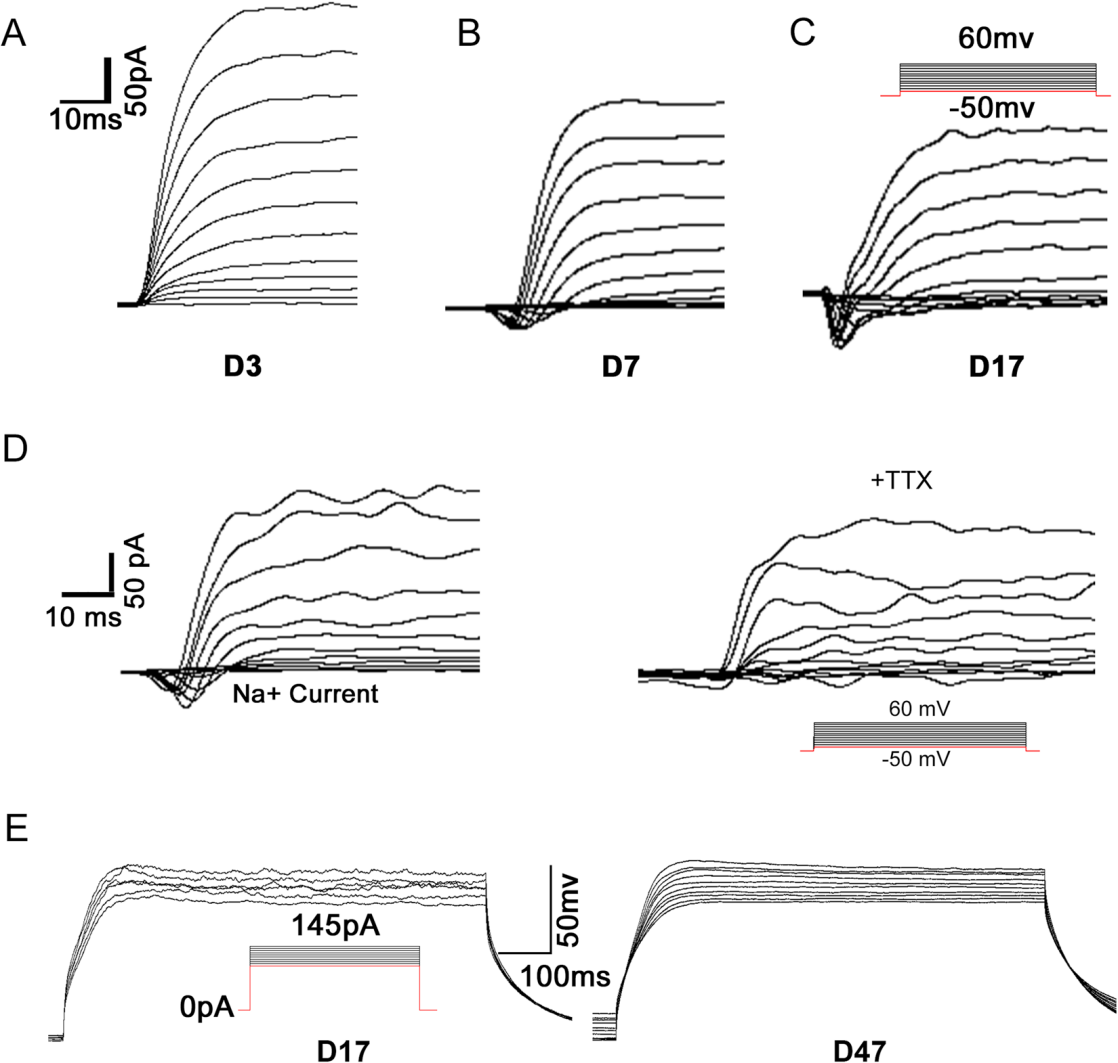
CiNCs possess partial electrophysiological properties. (**A**–**C**) Representative recordings of voltage-gated ion channels from a neuron of CiNCs. An outward current was observed as early as 3 days after chemical treatment (**A**) and an inward current was observed as as early as 7 days after chemical treatment (**B**); typical inward current and outward current was observed at day 17 (**C**). (**D**) The fast-inward currents were completely blocked by tetrodotoxin (TTX). (**E**) CiNCs failed to firing of action potentials at day 17 or day 47.

The maturity and function of CiNCs was further analyzed using the whole-cell patch-clamp recording. The results showed that these cells possessed partial electrophysiological properties. An outward current was observed 3 days after inputted CAYTFVB (Fig. 4A), and the inward current was observed at day 7 (Fig. 4B). More significant inward current and the outward current was observed at day 17 (Fig. 4C). The fast-inward current was completely blocked by a sodium channel antagonist tetrodotoxin (TTX) (Fig. 4D). However, these CiNCs still failed from firing action potentials at day 17 (Fig. 4E). Furthermore, patch clamp measurement was performed in the later stage at day 47, the results were similar like day 17, still no obvious action potential observed (Fig. 4E).

## Discussion

This study has established a fast and noninvasive approach that can convert human urine cells into neurons directly, without introducing the transcription factors or miRNAs. Compare with previous study, this approach used noninvasively collected cells and the typical neuronal morphology can be observed as early as 3 days. With the combination of seven small molecules of CHIR99021, A8301, Y27632, TTNPB, Forskolin, VPA, and NaB, urine cells could be converted into neurons within a week.

Cell transdifferentiation, which directly switches one type of differentiated cell into another cell type, needs to overcome cell identity barriers that keep the starting cell from converting into another identity^31^. To generate the desired cell type, the routine stability of the source cells needs to be disturbed. Small molecules that target specific signaling pathways, epigenetic processes and other cellular processes could manipulate cell fate changes, which make them powerful tools for inducing cell direct lineage reprogramming.

Wnt signaling activator and TGF-β signaling inhibitor are critical for inducing fibroblasts or astrocytes into neurons in chemical induction protocols^16,19,20,22^. A8301 is potent antagonist for all three subtypes of TGFβ RI including ALK-5, ALK-4 and ALK-7. CHIR99021 is a GSK3β inhibitor; and GSK3β has inhibitory role for canonical Wnt signaling through phosphorylation and degradation of β catenin. Upon Wnt binding to Frizzled and its co-receptor LRP5/6, it leads to the movement of GSK3β/APC/Axin complex to the membrane and phosphorylation of LRP5/6. Phosphorylated LRP5/6 blocks GSK3β mediated β catenin degradation. Thus, inhibition of GSK3β by CHIR99021 leads to the activation of Wnt signaling.

In order to reprogram urine cells into neurons, other signaling pathways also needs to be considered. We applied the RhoA inhibitor Y27632 in our formula. RhoA is a negative regulator for neuronal differentiation and neurites growth. Actin stability controlled by RhoA plays important role in the process of the elongation and branch of neurite^33^. In contrast, the positive neurites growth regulator such as Y27632, could induce the elongation and branch of neurite during neurogenesis and neuronal differentiation, by inhibiting RhoA^33^, and also enhance the survival of pluripotent stem cells and neurons^34,35^.

Retinoic acid (RA) induces neurogenesis and neuronal differentiation through activating RA receptors(RARs)^36–38^. The disruption of RA signal lead to the result of the decreased numbers of newborn neurons^39,40^. It was also reported that RAR signaling is beneficial for neuronal transdifferentiation. For example, An agonist of RA pathways, TTNPB, combined with the other 8 small molecules can convert human astrocytes into functional neurons^20^.

To further improve the survivability and maturation after the conversion, one of the methods was combining cAMP signaling activator Forskolin with the bone morphogenetic protein signaling inhibitor dorsomorphin^12^.

Cell fate conversion was accompanied by epigenetic changes, and epigenetic modulators can facilitate cell fate conversion by modifying chromatin structure and allowing the changes to occur. In this study, the HDAC inhibitors VPA and NaB were used as epigenetic modulators to facilitate the conversion process. NaB is a more potent HDAC inhibitor cause hyperacetylation of histones and we added sodium butyrate (NaB, 0.1 mM) for making the epigenetic modification more profound to lead to transdifferentiation.

Although the precise mechanism of this chemical induced reprogramming has not been elucidated, this study showed that activation of various signaling pathways, including Wnt, RA, and cAMP, and repression of ROCK and TGF-β pathways were essential to convert neurons from hUCs using protocol developed in this study. The small-molecule cocktail CAYTFVB was sufficient to activate the expression of neuronal master genes, which determine the specific cell identities of neurons and erase the initial cells specific gene expression.

Most of the transduced neurons died 16 days after input CAYTFVB, suggesting that the chemical cocktails may be incompatible with neuronal survival under these conditions. It has been reported that longer exposure to VPA increases cell death^20^. With the removal of VPA and NaB but added the small molecule VC in the medium 7 days after adding in CAYTFVB, higher percentage of survived neurons observed. This indicated VC can promote the survival and maturation of the CAYTFVB induced neurons, by protecting the cells from oxidative stress.

Cells in different brain regions have been targeted by various neurogenic fate determinants. In this study, CAYTFVB shown ability to convert hUCs to glutamatergic neurons. The key factors of this cocktail have potential to instruct the cell fate in the reprogramming process based on transcription factors. However, this protocol still insufficient to generate neuronal subtypes other than a glutamatergic neuron, which might be a valuable topic to be studied in the future.

Moreover, the source of cells was an important factor in direct neuronal reprogramming. Different source cell type implies different cellular contexts, such as chromatin, proteome or metabolome. Initial attempt reprogramming was based on an assumption that cells derived from the same lineage or the same germ layer with the target cell type might be easier to be converted. Thus, the cells related to neurons are chosen to be the source for neuronal conversion. Previous studies reported astrocytes sharing a common origin with neurons have been converted into functional neurons, such as human fibroblasts, which directly converted into neurons by small molecules^19^. However, invasive surgical biopsy procedures were necessary to isolate human astrocytes or fibroblasts. In comparison, human urine cells were an unlimited autologous cell source that can be easily isolated without any invasive surgical biopsy procedures. This may reduce the risk and cost of potential clinical applications in future.

In summary, the design strategy for our seven small molecules (CAYTFVB) cocktail is included four mechanistic roots: 1) To inhibit ROCKs by Y-27632, reducing the stress fiber network form and remodelling the cytoskeleton more flexible for changes; the Y-27632 compound is an ATP-competitive inhibitor of ROCK-I and ROCK-II, with Ki (Kinase activity inhibited by 50%) of 220 nM and 300 nM for ROCK-I and ROCK-II, respectively. 2) To promote cell survival ability and protect from apoptosis; TGF-β-mediated growth inhibition is the major blockage for cell culture *in vitro*, the chemical A-83-01 is a potent antagonist for TGF-β RI ALK4/5/7, the inhibition of TGF-β promotes cell proliferation and also the neurogenesis; meanwhile, increasing intracellular levels of cAMP by Forskolin treatment provides neurotrophic activity, against oxidative stress and survivability; 3) Inducing epigenetic modification by inhibition of HDACs relieve HDAC-dependent transcriptional repression; the combination of ROCK inhibitors (Valproic acid and Sodium Butyrate) is the main force for this job; 4) To activate neuronal lineage driving pathways including Wnt, RAR and cAMP-dependent PKA pathways; CHIR 99021 inhibits GSK3 and activates Wnt signaling, TTNPB activates RARs, and Forskolin is a potent adenylate cyclase activator and has a neurotrophic activity. With the mechanistic chemical combination, transdifferentiation of hUCs into hNCs was achieved with a relative higher efficiency.

## Methods

### Ethical statement

The cell lines used in this report were approved by the Ethics Committee of Guangzhou Institutes of Biomedicine and Health, Chinese Academy of Sciences. In addition, all experiments were performed in accordance with the ISSCR guidelines.

### Human urine cells isolation and culture

Four donors were recruited for urine samples with informed consent based on IRB approval (no. GIBH-IRB02-2009002) at Guangzhou Institutes of Biomedicine and Health (GIBH). The procedures and purposes for isolating urine cells and generating neurons were explained to the donors in detail, and questions, if any, were answered in full. We then obtained a formal signed consent form. The procedures for isolating human urine cells and generating neurons were performed as approved. hUCs were isolated from the urine of healthy donor as previously described^27^. Briefly, about 250 ml of fresh, clean-catch urine samples were collected from donor and transferred to 50 ml tube. The samples were centrifuged for 10 min at 400 *g*; the supernatant was discarded, and the pellet was resuspended. Then, 10 ml of washing buffer was added and centrifuged at 400 *g* for about 10 min. After centrifugation, the supernatant was discarded, and 1 ml of urine cell medium was added to suspend the cell pellet, which was then transferred into gelatin-coated six-well plates. Urine cell medium (2 ml, supplement with Primocin) was added, and the cells were incubated at 37 °C. The culture was then added with 1 ml of primary medium for subsequent 3 days; 5–7 days after plating, the medium was aspirated, and 2 ml urine cell medium was added. The medium was changed every 3 days with fresh one. The urine cell medium was changed every 2 days, and incubation was continued until the cells reach 80–90% density. When the cultured urine cells become dense enough for passaging, all the cells were split onto a new well of 100 mm plate for further expansion; this was considered passage 1 (P1). hUCs were used at passage 4–5 (P4–P5) for the following experiments.

### Generation of CiNCs

Small molecules were dissolved and diluted in DMSO according to the manufacturer’s instructions. Initial hUCs were seeded onto Matrigel (Corning, USA)-coated culture plates (20000–30000 cells/well in 24-well plates, Corning) and cultured in urine cell medium. After 48 h, a neuronal induction medium was used for culture. The neuronal induction medium contained NM3 basic neuronal induction medium supplemented with 10 μM Y27632, 5 μM A8301, 3 μM CHIR99021, 1 μM TTNPB, 5 μM Forskolin, 0.5 mM VPA, and 0.1 mM NaB. The neuronal induction medium was changed every 3 days. At day 7, cells were switched to neuronal maturation medium with the chemical cocktail CAYTF+ 0.2 mM VC. At day 17, induced neuronal cells were changed to culture in neuron medium to promote neuron survival and maturation. The detailed information of the protocol is included in Supplementary Experimental Procedures, as well as the medium and chemicals used. The detailed information of hUCs used is listed in Supplementary Table S2.

### Immunofluorescence staining

Cells were fixed with 4% paraformaldehyde for 50 min and washed with PBS. Then, cells were blocked and permeabilized with blocking solution (PBS containing 5% fetal bovine serum (FBS) and 0.2% Triton X-100) for 1 h at room temperature. The cells were probed with appropriate dilutions of primary antibodies in blocking solution at 4 °C overnight. Next, the cells were incubated with secondary antibodies for 1 h at room temperature. The cells were washed with PBS and stained with DAPI (Sigma) for 5 min, and then observed using a fluorescence microscope (Zeiss 710 NLO). Antibodies used in this study are listed in Supplementary Table S3.

### Real-time PCR analysis

Total RNA was isolated using RNA Pure total RNA micro kit (Magen) per manufacturer’s instructions. Real-time PCR (RT-PCR) was performed using a Bio-Rad CFX96 sequence detection system and SYBR green I dye reagent (Bio-Rad). The relative gene expression was analyzed using the 2^−ΔΔCt^ method. Target gene expression was normalized to the expression level of GAPDH. Primers used in this study were listed in Supplementary Table S4.

### Electrophysiology analysis

Whole-cell patch-clamp recordings were obtained at room temperature. Signals were amplified with a MultiClamp 700B amplifier, digitized with a Digidata 1440, and acquired with pClamp 10 software (Molecular Devices, USA). The filter was Bessel filter, cutting frequency 3000 Hz. The bath solution contained (in mM): 127 NaCl, 5 KCl, 2 MgCl_2_, 2 CaCl_2_, 10 HEPES, and 12 glucose (pH 7.4 with NaOH, 300 osmol/L). Patch pipettes with resistance between 8–10 MΩ were pulled from borosilicate glass (WPI, USA) with a Sutter P97 puller (Sutter, USA). Pipettes were filled with solutions containing (in mM): 145 K-gluconate, 0.2 EGTA, 10 HEPES, 5 NaCl, 1 MgCl_2_, 4 Mg-ATP, and 0.3 Na-GTP (pH 7.2 with KOH, 285 osmol/L). In recording GABA activated currents and spontaneous inhibitory synaptic currents, CsCl-based solution was used to obtain inward chloride currents (in mM):130 CsCl, 4 NaCl, 1 MgCl_2_, 10 HEPES, 5 EGTA, 2 QX-314, 2 MgATP, and 0.2 Na-GTP (pH 7.2 with CsOH, 285 osmol/L).

### Statistical analysis

For all tests, P < 0.05 was considered statistically significant. All data is represented at the mean +/− standard error of the mean (SEM).

## Supplementary information

Supplemental information

## Data Availability

The data that support the findings of this study are available from the corresponding author upon reasonable request.
