# Supplementary material for: Direct Conversion of Human Urine Cells to Neurons by Small Molecules
Source: Sci Rep. 2019 Nov 13;9:16707. doi: 10.1038/s41598-019-53007-6 (PMC6854089; doi:10.1038/s41598-019-53007-6)
Supplement: Supplementary file 1 — Supplemental information [file 41598_2019_53007_MOESM1_ESM.pdf]

Scientific Reports

Supplementary Information

Direct Conversion of Human Urine Cells to Neurons by Small Molecules

Guosheng Xu [a,b,c,d,f], Feima Wu [a,b,c,d], Xiaotong Gu [a], Jiaye Zhang[a], Kai You [a,b,c], Yan Chen [a,b,c], Anteneh Getachew [a,b,c,d], Yuanqi Zhuang [a,b,c,d], Xiaofen Zhong [a,b,c,d], Zuoxian Lin [a], Dongsheng Guo [a,b,c,d], Fan Yang [a,b,c], Tingcai Pan [a,b,c,d], Hongcheng Wei [g], Yin-xiong Li [a,b,c,d,e,h]

[a] Institute of Public Health, Guangzhou Institutes of Biomedicine and Health, Chinese Academy of Sciences, Guangzhou, China

[b] Guangdong Provincial Key Laboratory of Biocomputing, Guangzhou Institutes of Biomedicine and Health, Chinese Academy of Sciences, Guangzhou, China

[c] Key Laboratory of Regenerative Biology, South China Institute for Stem Cell Biology and Regenerative Medicine, Guangzhou Institutes of Biomedicine and Health, Chinese Academy of Sciences, Guangzhou, China

[d] University of Chinese Academy of Sciences, Beijing, China

[e] Guangdong Provincial Key Laboratory of Stem Cell and Regenerative Medicine, Guangzhou, China

[f] Guangzhou Blood Center, Guangzhou, China

[g] Department of Gastroenterology, The First Affiliated Hospital of Jinan University, Guangzhou, China

[h] Guangzhou Regenerative Medicine and Health Guangdong Laboratory, Guangzhou, China

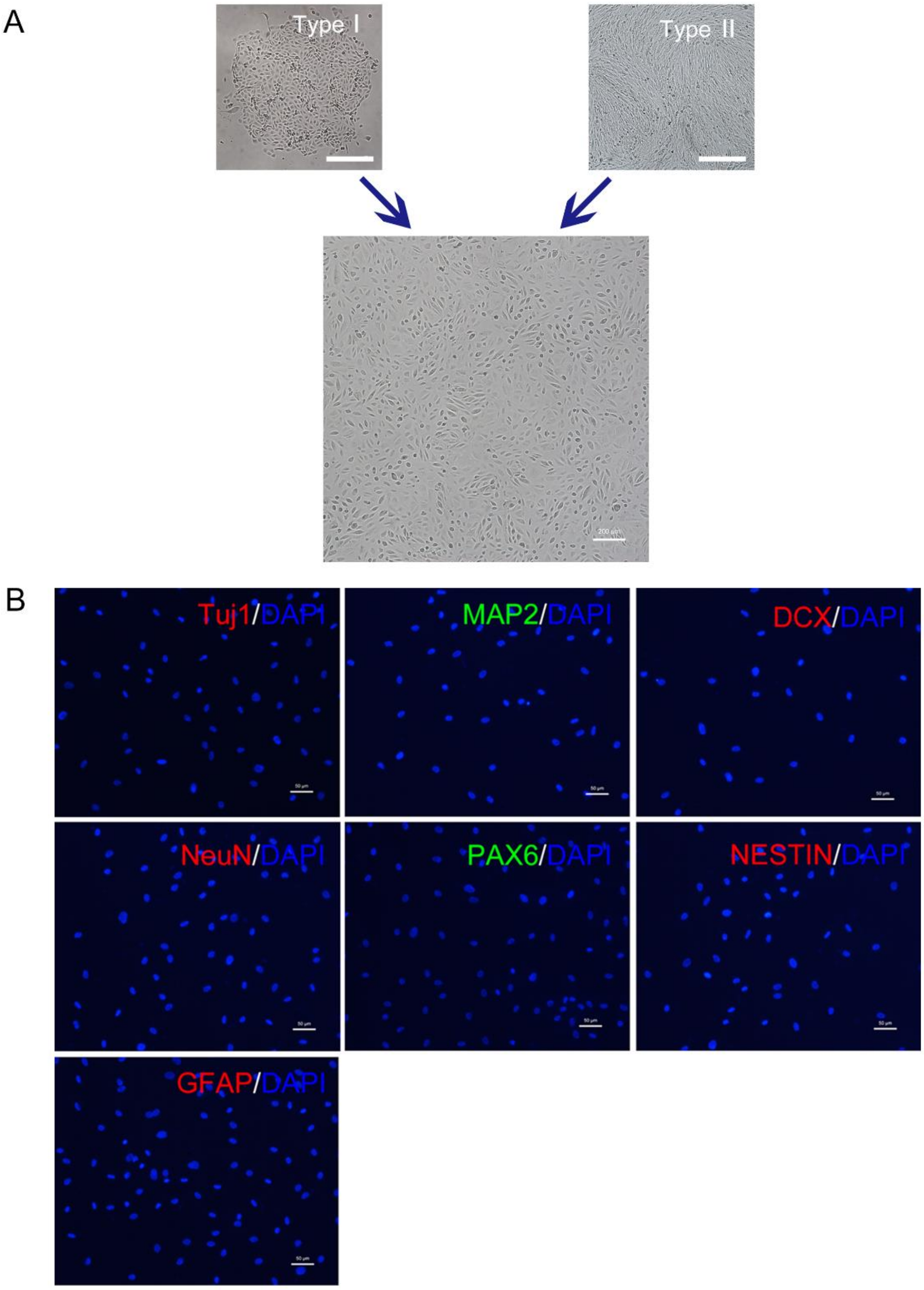

Supplementary Figure S1. Isolation and characterization of human urine cells

(A) Bright-field image of isolated human urine cells(P0). The urine cells exhibited two types of main morphologies from different colonies in the same petri dish, they were mixed together after culturing for 2 weeks. Scale bars, 200 μm.

(B) Immunofluorescence staining of human urine cells showed that neither NSCs nor neurons were found in the hUCs. Scale bars, 50 μm.

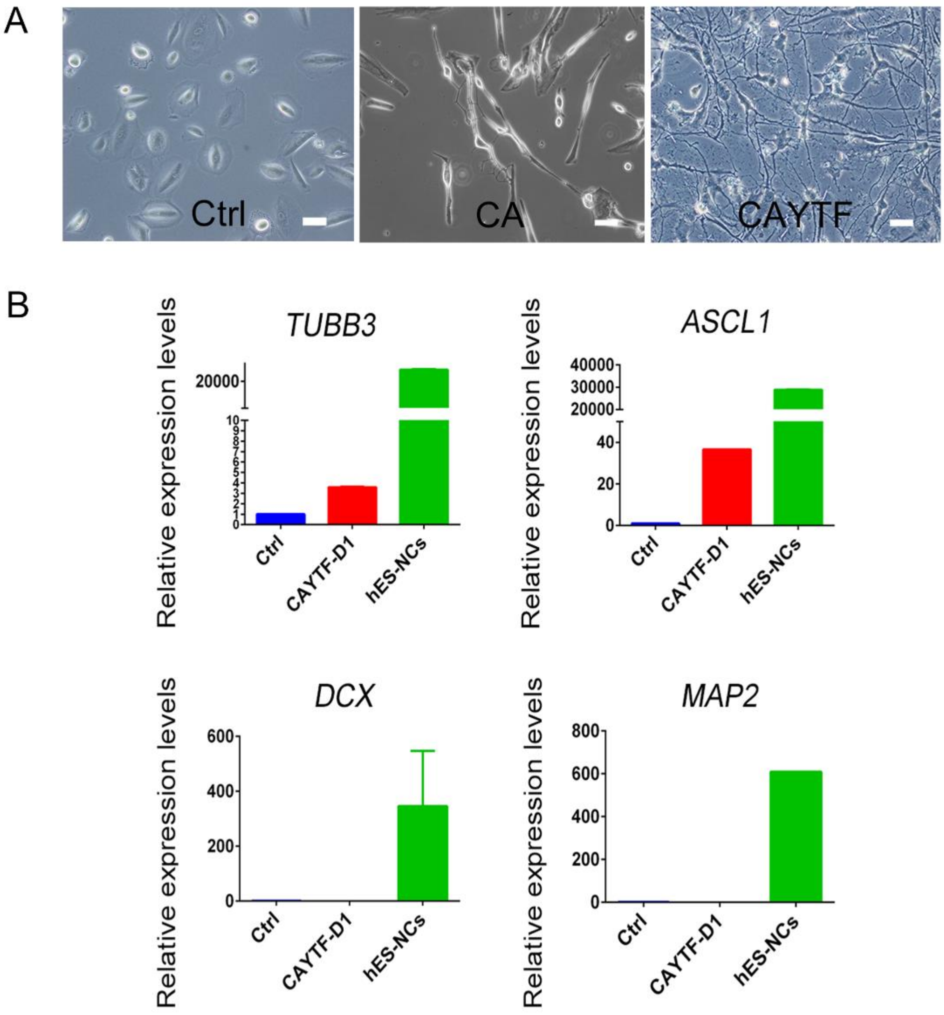

Supplementary Figure S2. CAYTF induce the transdifferention of the hUCs to neuronal fate

(A) Bright-field image of control hUCs (left) and CA treatment (middle) or CAYTF treatment at day 10 (right). C, CHIR99021; A, A8301; Y, Y-27632; T, TTNPB; F, Forskolin. Scale bars, 100 µm.

(B) Upregulation of neuronal master gene *ASCL1* and *TUBB3*. hUCs were treated with CAYTF for 1 day. hUCs (no treatment) were used as negative control and all sample data was normalized to that of hUCs, which was considered as 1. hES derived neurons were used as positive control. Data of three independent experiments were shown as means ± SEM.

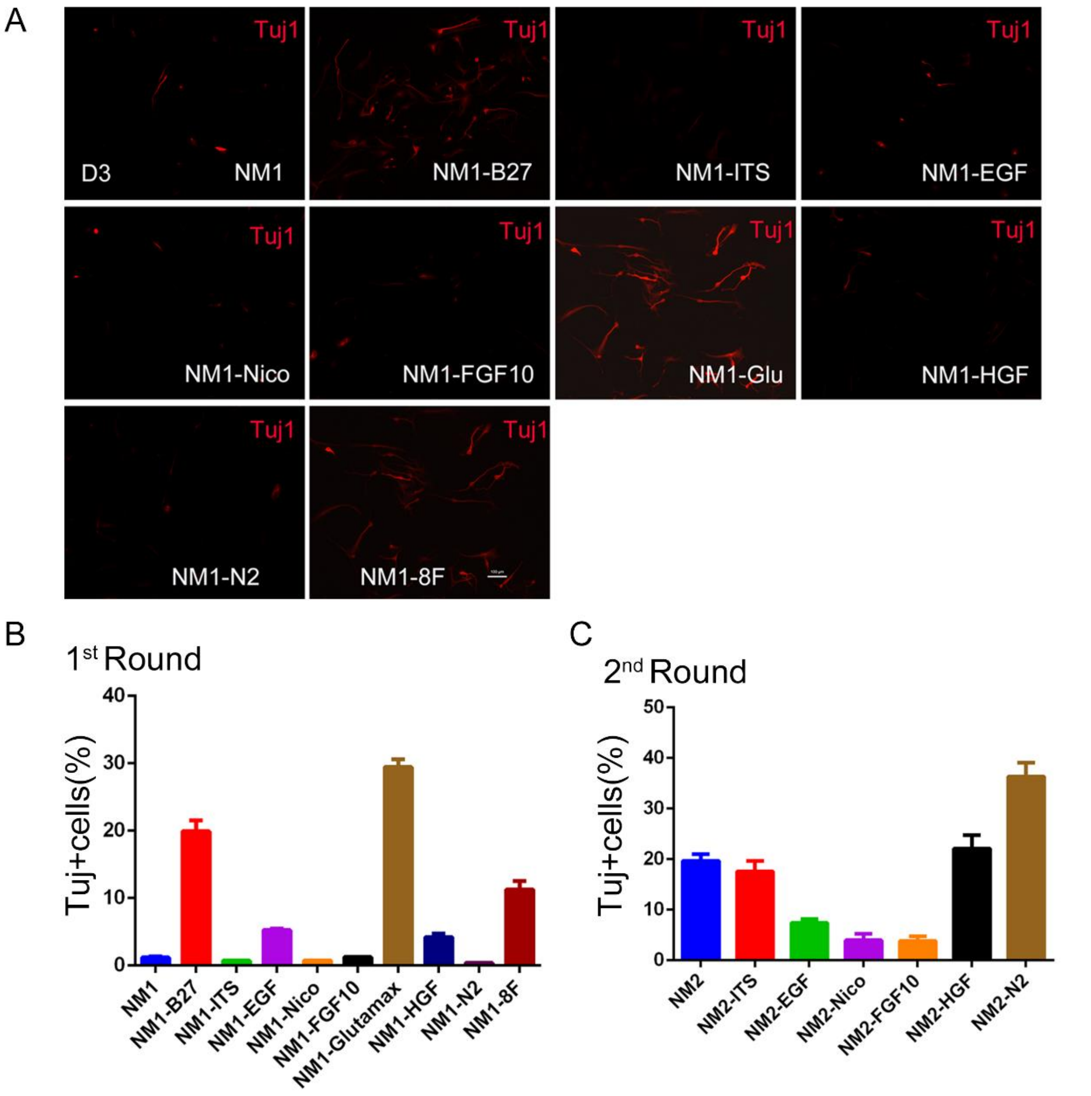

Supplementary Figure S3. Optimizing of the basic neuronal induction medium

(A) Immunofluorescence staining of induced cells showed the result of the first round of basic neuronal induction medium optimized. Immunofluorescence staining was performed at day 3. Scale bars, 100 μm.

(B) Percentages of Tuj^+ cells during first round of basic neuronal induction medium optimized. Immunofluorescence staining was performed at day 3. (means ± SEM, n = 20 random selected ×20 fields from triplicate samples)

(C) Percentages of Tuj^+ cells during second round of basic neuronal induction medium optimized. Immunofluorescence staining was performed at day 3. (means ± SEM, n = 20 random selected ×20 fields from triplicate samples)

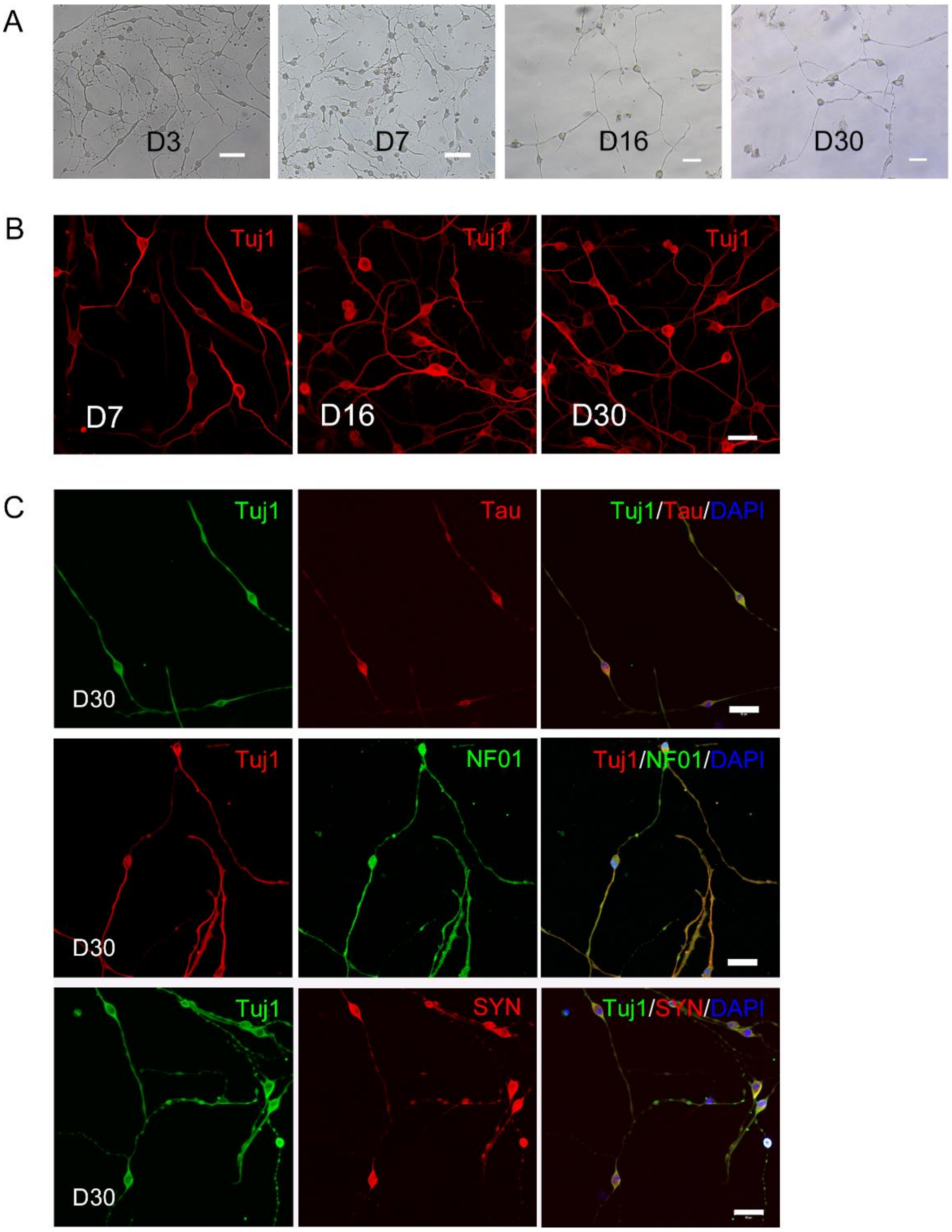

Supplementary Figure S4. The morphology changes and neuron markers expression of CiNCs in the whole induction process

(A) Bright-field image show the morphology changes of CiNCs in the whole induction process. The cells at day 3 and day 7 were cultured in induction medium containing the CAYTFVB. The medium was neuron maturation medium containing CAYTF+VC and extra neurotropic factors (BDNF, GDNF, and NT3) at day 16. At day 30, culture medium was neuron medium (without the small molecules). Scale bars, D3 and D7, 100 μm, D16 and D30, 50 μm.

(B) Immunofluorescent staining showed that neuron marker Tuj1 can be detected in the whole induction process. The cells at day 7 were cultured in induction medium containing the

CAYTFVB. The medium was neuron maturation medium containing CAYTF+VC and extra neurotropic factors (BDNF, GDNF, and NT3) at day 16. At day 30, culture medium was neuron medium (without the small molecules). Scale bars, 50 μm.

(C) Immunofluorescence staining showed that mature neuron markers Tau, NF-01 and SYN can be detected of CiNCs at day 30. Scale bars, 50 μm.

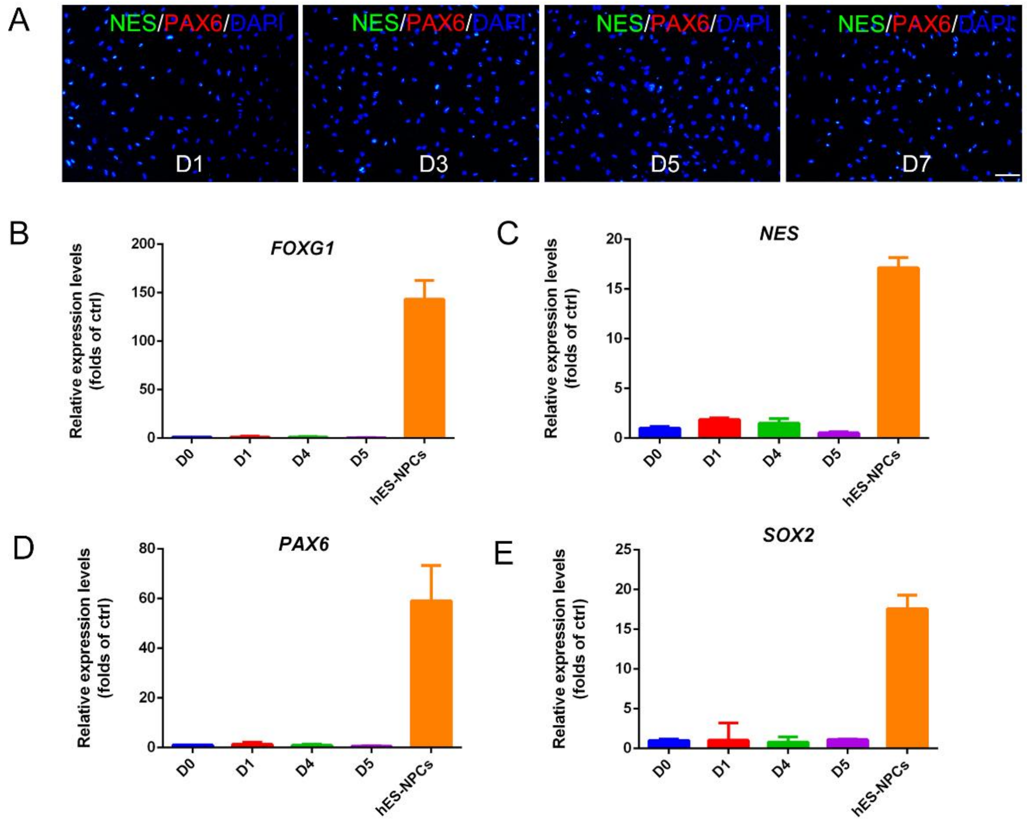

Supplementary Figure S5. Induction of CiNCs bypasses neural progenitor cell stage.

(A) Immunofluorescent staining showed that no detectable expression of neural progenitor cells specific markers during neuronal conversion. Scale bars, 100 μm.

(B-E) No expression of neural progenitor markers *FOXG1, NES, PAX6* or *SOX2* during neuronal conversion. All sample data are normalized to that of d0, which is considered as 1. hES derived neuronal progenitor cells(hES-NPCs) were used as positive control. Data of three independent experiments were shown as means ± SEM.

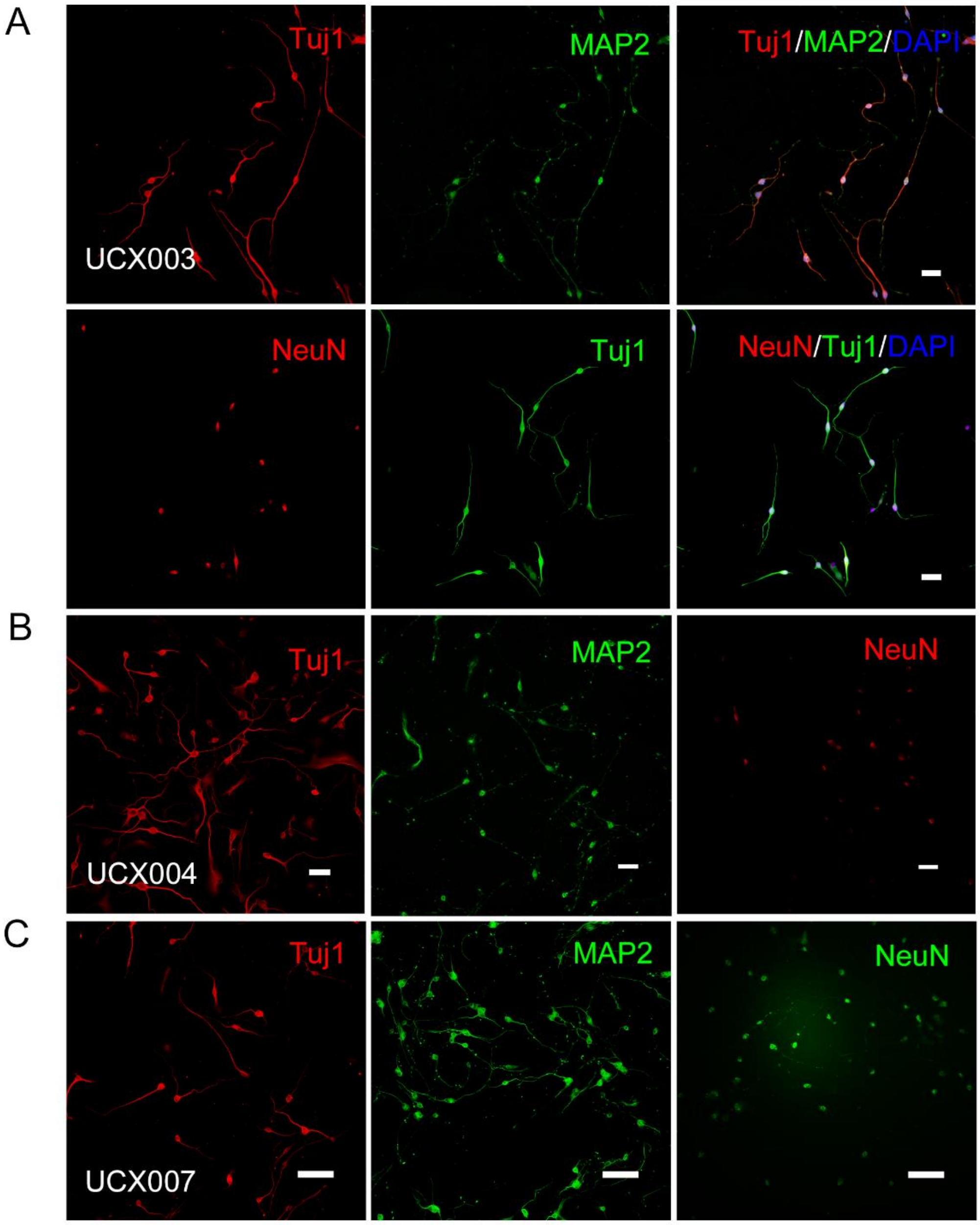

Supplementary Figure S6. Generation of CiNCs from hUCs derived from different donors by using the two-stage induction protocol

(A) Generation of CiNCs from human urine cell line UCX003. Cells were induced by the two-stage induction protocol. Immunofluorescent staining was performed at day 17. Scale bars, 100 μm.

(B) Generation of CiNCs from human urine cell line UCX004. Cells were induced by the two-stage induction protocol. Immunofluorescent staining was performed at day 17. Scale bars, 100 μm.

(C) Generation of CiNCs from human urine cell line UCX007. Cells were induced by the two-stage induction protocol. Immunofluorescent staining was performed at day 17. Scale bars, 100 μm.

Supplementary Table S1. Information of basic neuronal induction medium

NM1	NM2	NM3
Advanced DMEM/F12	Advanced DMEM/F12	Advanced DMEM/F12
1×B27	1×ITS	50ng/ml EGF
1×ITS	50ng/ml EGF	0.5mM Nico
50ng/ml EGF	0.5mM Nico	100ng/ml FGF10
0.5mM Nico	100ng/ml FGF10	
100ng/ml FGF10	20ng/ml HGF	
1×Glutamax	0.5% N2	
20ng/ml HGF		
0.5% N2		

Supplementary Table S2. Detailed information of human urine cells

Line	Passage	Cell type	Objects
UCX001	4-6	human urine cells	32, male
UCX003	4-6	human urine cells	25, female
UCX004	4-6	human urine cells	20, female
UCX007	4-6	human urine cells	30, male

Supplementary Table S3. Primary antibody list.

Antibodies	Source	Cat#	Dilution
Mouse anti-Tuj1	abCAM	AB78078	1:1000
Rabbit anti-Tuj1	abCAM	AB18207	1:1000
Mouse anti-MAP2	MILIPORE	MAB3418	1:1000
Rabbit anti-NeuN	MILIPORE	ABN78	1:500
Rabbit anti-Nestin	MILIPORE	ABD69	1:1000
Mouse anti-Pax6	abCAM	AB78545	1:1000
Rabbit anti-HB9	DSHB	81.5C10	1:500
Rabbit anti-TH	MILIPORE	AB152	1:500
Rabbit anti-GABA	SIGMA	A2052	1:500
Rabbit anti- Glutamate	SIGMA	G6642	1:500
Rabbit anti- Tau	abCAM	AB76128	1:500
Rabbit anti-DCX	abCAM	AB77450	1:1000
Rabbit anti-Synapsin I	Millipore	MABN894	1:500
Mouse anti-NF-H	abcam	ab7795	1:500
Mouse anti-GFAP	MILIPORE	MAB360	1:1000

Supplementary Table S4. Primers for qRT-PCR.

Primers	Forward	Reverse
SOX2	GCCGAGTGGAAACTTTTGTCG	GGCAGCGTGTACTTATCCTTCT
PAX6	TGGGCAGGTATTACGAGACTG	ACTCCCGCTTATACTGGGCTA
FOXG1	CCGCACCCGTCAATGACTT	CCGTCGTAAAACTTGGCAAAG
NES	CTGCTACCCTTGAGACACCTG	GGGCTCTGATCTCTGCATCTAC

TUBB3	GGCCAAGGGTCACTACACG	GCAGTCGCAGTTTTCACACTC
DCX	TTCAAGGGGATTGTGTACGCT	GTCAGACAGAGATCGCGTCAG
ASCL1	CGCGGCCAACAAGAAGATG	CGACGAGTAGGATGAGACCG
MAP2	CTCAGCACCGCTAACAGAGG	CATTGGCGCTTCGGACAAG
PSD95	TCGGTGACGACCCATCCAT	GCACGTCCACTTCATTTACAAAC
POU3F2	CGGCGGATCAAACTGGGATTT	TTGCGCTGCGATCTTGTCTAT
NEUROND1	GTCTCCTTCGTTCAGACGCTT	AAAGTCCGAGGATTGAGTTGC
NGN2	AGGAAGAGGACGTGTTAGTGC	GCAATCGTGTACCAGACCCAG
MYT1L	CTCGGCAAAATCGCTGAGGAT	TCCAGACTATTGGAGGTATTGCT

Supplementary Experimental Procedures

Cell culture

Four human urine cells (UCX001, UCX003, UCX004, UCX007) were isolated from the urine of healthy donor. The detail information of these human urine cells was listed in Supplementary Table S1. Human urine cells were maintained in urine cell medium consisting of a 1:1 mixture of DMEM/F12 culture medium supplemented with 10% of FBS (FBS, Gbico), 0.1 mM non-essential amino acids (NEAA, Gbico), 1 mM GlutaMAX (Life Technologies), and SingleQuot Kit CC-4127 REGM (Lonza) and penicillin/streptomycin at 37°C with 5% CO2. The urine cell medium was changed every two days.

Generation of CiNCs

Reagents:

CHIR99021, 3 μM (Selleck, S2924, 6 mM stock in DMSO);

A8301, 5μM (Sigma, SML0788, 10 mM stock in DMSO);

Y-27632, 10μM (Selleck, S1049, 10 mM stock in DMSO);

TTNPB, 1μM (Selleck, S4627, 5 mM stock in DMSO);

Forskolin, 5μM (selleck, S2449, 50 mM stock in DMSO);

VPA, 0.5 mM (merck, PHR1061, 1 M stock in H_2O);

NaB, 0.1 mM (selleck, A510838, 100mM stock in DMSO);

Vitamin C, 0.2 mM (Sigma, 200mM stock in H_2O);

FGF-10, (PeproTech, AF100-26, 100ug/ml stock in H_2O);

cAMP, 100 μM (Sigma-Aldrich, D0260, 100 mM stock in H_2O);

bFGF, 20 ng/ml (Invitrogen, PHG0024, 100 μg/ml stock in H_2O);

BDNF, 20 ng/ml (PeproTech, 450-02, 100 μg/ml stock in H_2O);

GDNF, 20 ng/ml (PeproTech, 450-10, 100 μg/ml stock in H_2O);

NT3, 20 ng/ml (PeproTech, 450-03, 100 μg/ml stock in H_2O).

Medium:

urine cells medium: 1:1 mixture of DMEM/F12 culture medium supplemented with 10% of FBS (FBS, Gbico), 0.1 mM non-essential amino acids (NEAA, Gbico), 1 mM GlutaMAX (Life Technologies), and SingleQuot Kit CC-4127 REGM (Lonza) and penicillin/streptomycin.

Neuronal induction medium: (Advanced DMEM/F12, Life Technologies, 11330-032): (Neurobasal, Life Technologies, 21103-049) (1:1), 1% ITS (Invitrogen, 17502048), 0.5 mM Nicotinamide (Stemcell, 07154), and 50 ng/ml EGF and penicillin/streptomycin.

Neuronal maturation medium: Advanced DMEM: Neurobasal (1:1), 0.5% N-2, 1% B-27, 100 μM cAMP, 20 ng/ml bFGF, 20 ng/ml BDNF, 20 ng/ml GDNF, 20 ng/ml NT3 and penicillin/streptomycin.

Neuron medium: DMEM/F12: Neurobasal (1:1), 0.5% N-2, 1% B-27, 20 ng/ml BDNF, 20 ng/ml GDNF, 20 ng/ml NT3 and penicillin/streptomycin.

Procedure:

1. 24-wells or 6-wells plates with or without coverslips were coated with 1% matrigel for more than half hour.
2. Initial urine cells were seeded onto gelatin-coated culture plates (20,000-30,000 cells/well in 24-well plates) and cultured in urine cells medium for 48 hours.
3. The cell confluence was about 40-60%, and the cells were transferred into neuronal induction medium with chemical cocktails CAYTFVB. The concentration of chemicals used is: CHIR99021, 3 μM; A8301, 5μM; Y-27632, 10μM; TTNPB, 1μM; Forskolin, 5μM; VPA, 0.5 mM; NaB, 0.1 mM.
4. Medium containing chemical compounds was changed every two days.
5. After 7 days, cells were switched to neuronal maturation medium with CAYTF+VC

(CHIR99021, 3 μM; A8301, 5μM; Y-27632, 10μM; TTNPB, 1μM; Forskolin, 5μM; VPA, 0.5 mM; NaB, 0.1 mM; VC, 0.2 mM.)

6. Maturation medium was half-changed every three days.

7. After 17 days, induced neuronal cells were cultured in neuron medium to promote neuron survival and maturation.

8. Half-change the neuron medium every three days until the cells are subjected to further analysis.
